# PTEN, ERG, SPINK1, and TFF3 Status and Relationship in a Prostate Cancer Cohort from Jordanian Arab Population

**DOI:** 10.3390/medicina60010174

**Published:** 2024-01-18

**Authors:** Samir Al Bashir, Mohammed S. Alorjani, Khalid Kheirallah, Mohammad Al Hamad, Husam K. Haddad, Ahmad Al-Dwairy, Baha A. Bani-Fawwaz, Najla Aldaoud, Omar Halalsheh, Saddam Amawi, Ismail I. Matalka

**Affiliations:** 1 Department of Pathology and Microbiology, Faculty of Medicine, Jordan University of Science and Technology, Irbid 22110, Jordan; msalorjani@just.edu.jo (M.S.A.); nhaldaoud2@just.edu.jo (N.A.); imatalka@just.edu.jo (I.I.M.); 2 Department of Public Health and Community Medicine, Faculty of Medicine, Jordan University of Science and Technology, Irbid 22110, Jordan; kkheiral@gmail.com; 3 Department of Pathology, College of Medicine, Imam Abdulrahman Bin Faisal University, Dammam 31441, Saudi Arabia; mhamad@iau.edu.sa; 4 Department of Pathology and Laboratory Medicine, Ministry of Health, Amman 11118, Jordan; hkhaddad06@gmail.com; 5 Medstar-Georgetown Washington Hospital Center, Georgetown University, Washington, DC 20057, USA; dwairy_ahmad@yahoo.com; 6 Gastroenterology and Hepatology Department, Adventhealth, Orlando, FL 32804, USA; bahaadamen@hotmail.com; 7 Department of General Surgery and Urology, Faculty of Medicine, Jordan University of Science and Technology, Irbid 22110, Jordan; omhalalsheh@just.edu.jo; 8 Johns Hopkins Aramco Health Centre, Al Mubarraz 36423, Saudi Arabia; saddamamawi@yahoo.com; 9 College of Medicine, Ras Al-Khaimah (RAK) Medical and Health Sciences University, Ras Al-Khaimah 11172, United Arab Emirates

**Keywords:** prostate cancer, PTEN, ERG, SPINK1, TFF3

## Abstract

*Background and Objectives*: Prognostic biomarkers in prostate cancer (PCa) include PTEN, ERG, SPINK1, and TFF3. Their relationships and patterns of expression in PCa in developing countries, including Jordan, have not yet been investigated. *Materials and Methods*: A tissue microarray (TMA) of PCa patients was taken from paraffin-embedded tissue blocks for 130 patients. PTEN, ERG, SPINK1, and TFF3 expression profiles were examined using immunohistochemistry (IHC) and correlated with each other and other clinicopathological factors. *Results*: PTEN loss of any degree was observed in 42.9% of PCa cases. ERG and TFF3 were expressed in 59.3% and 46.5% of PCa cases, respectively. SPINK1 expression was observed in 6 out of 104 PCa cases (5.4%). Among all PCa cases (*n* = 104), 3.8% (*n* = 4) showed SPINK1+/ERG+ phenotype, 1.9% (*n* = 2) showed SPINK1+/ERG- phenotype, 56.7% (*n* = 59) showed SPINK1-/ERG+ phenotype, and 37.5% showed SPINK1-/ERG- phenotype (*n* = 39). Among ERG positive cases (*n* = 63), 6.3% were SPINK1 positive. Among SPINK1 positive cases (*n* = 6), 66.7% were ERG positive. SPINK1 expression was predominantly observed in a subgroup of cancers that expressed TFF3 (6/6). Additionally, a statistically significant loss of PTEN expression was observed from Gleason Score 6 (GS6) (Grade Group 1 (GG1)) to GS9-10 (GG5); (*p*-value 0.019). *Conclusions*: This is the first study to look at the status of the PTEN, ERG, SPINK1, and TFF3 genes in a Jordanian Arab population. Loss of PTEN has been linked to more aggressive prostate cancer with high GSs/GGs. SPINK1 expression was predominantly observed in a subgroup of cancers that expressed TFF3. Our results call for screening these biomarkers for grading and molecular subtyping of the disease.

## 1. Introduction

Prostatic carcinoma (PCa) is the fourth most common malignancy in both sexes combined, with an estimated rate of 7.3% after female breast (11.7%), lung (11.4%), and colorectal (10.0%) [1]. PCa is the eighth-leading cause of cancer death with an estimated rate of 3.8% surpassed by lung (18.0%), colorectal (9.4%), liver (8.3%), stomach (7.7%), female breast (6.9%), esophagus (5.5%), and pancreas (3.8%) [1]. In Jordan, PCa is the fourth most common cancer among the male population, with an incidence rate of 15.9 per 100,000 and a mortality rate of 8.3 per 100,000 [2]. It is known that multiple genetic alterations are displayed in the prostate gland, leading to the development of PCa. Studying these genetic changes could be challenging [3,4,5,6]. Phosphatase and TENsin homolog (PTEN), Erythroblast transformation-specific–related gene (ERG), Serine protease inhibitor Kazal-type 1 (SPINK1), and Trefoil Factor 3 (TFF3) have been identified as important biomarkers for prostate cancer.

PTEN, a tumor suppressor gene located on chromosome 10q23, plays a major role in cell growth, survival, and migration by inhibiting the PI3K/AKT signaling pathway [7]. Loss or inactivation of PTEN has been observed in various cancers, including PCa and breast cancer [7]. ERG is an oncogene encoding for a protein that functions as a transcriptional regulator. It is engaged in multiple translocations such as TMPSSR2-ERG and NDRG1-ERG resulting in its overexpression in PCa [8]. ERG fusion proteins have been shown to promote prostate cancer cell proliferation and invasion [9]. An association between PTEN loss and ERG gene rearrangements was also observed in a subset of PCa, which is the most common genetic abnormality in PCa [6,10]. In 1948, Kazal et al. detected SPINK1 protein in the urine of an ovarian cancer patient. It is a trypsin inhibitor that was later found to be expressed in pancreatic acinar cells and various diseases [11,12,13,14,15]. It was also proposed that the SPINK1 protein is a marker for PCa that lacks ETS gene fusions. Furthermore, it was noted that SPINK1 expression was associated with poor prognosis in PCa patients [16]. However, other studies did not find SPINK1 expression to be a useful prognostic marker [17,18,19,20]. TFF3 is a peptide that is secreted by intestinal goblet cells and has been found in multiple organs, diseases, and tumors [21,22,23,24,25]. Furthermore, TFF3 has been found to be a leading factor in cancer, contributing to cellular proliferation, evasion of apoptosis, tumor invasion, and the formation of new blood vessels [26].

In 2013, Park et al. discovered that a coexpression of ERG and TFF3 by immunohistochemistry (IHC) is relevant for PCa detection, with a sensitivity of 76% and a specificity of 96% [23]. However, TFF3 is negatively correlated with TMPRSS2-ERG status in specimens collected from surgically treated PCa patients [27]. Additionally, other studies revealed that ERG, TFF3, and SPINK1 are associated with increased cell motility and/or aggressiveness of PCa, suggesting that the aforementioned gene products potentially play a role in PCa development and/or progression [16,27,28]. This study was conducted to highlight the importance of PTEN, ERG, SPINK1, and TFF3 as a biomarker for PCa grading and molecular subtyping.

## 2. Material and Methods

### 2.1. Specimen Collection

A total of 130 PCa specimens were collected from the archives of King Abdullah University Hospital (KAUH) in Irbid, Jordan, spanning a period between 2005 and 2018. PCa cases included 54 radical prostatectomies (RP) and 76 transurethral resections of prostate (TURP). Using a fully automated tissue microarrayer (TMA Master II 3DHISTECH), three TMA blocks were generated from this cohort. Each block was put together blindly without any prior knowledge of clinical or pathological staging. From the paraffin-embedded tissue blocks comprising benign and PCa, one to nine cores (average 3.3), 0.6 mm in diameter, were sampled from each case.

Following construction, 4 μm sections were cut and stained with hematoxylin and eosin on the initial slides to confirm the histological diagnosis and Gleason Score (GS) and Grade Group (GG)/International Society of Urological Pathology (ISUP) grading. Three pathologists (SAB, NA, and MSA) independently and blindly assessed all cases. According to the criteria of the 2005 and 2014 ISUP consensus conferences [29,30], we sampled both Gleason patterns originally present in RP or TURP to reflect the GS and GG from each patient. The ISUP grading group score was divided into two groups: low-risk (GG 1–3) and high-risk (GG 4–5).

From patients’ medical records, clinical data including age, preoperative prostate-specific antigen (PSA), postoperative PSA, and biochemical recurrence were obtained. A case was considered to have a biochemical recurrence if there was an increase in PSA level above 0.2 ng/mL [31]. All PCa cases were diagnosed as prostatic acinar adenocarcinoma. No mixed (acinar/ductal) or ductal adenocarcinoma cases were included. The study was approved by the Institutional Review Board of Jordan University of Science and Technology.

### 2.2. Immunohistochemistry (IHC) Evaluation

IHC was conducted on 4 -μm sections of the TMA for PTEN, ERG, SPINK1, and TFF3 using a Dako Autostainer-Plus (Dako, Denmark) according to the manufacturer’s guidelines. In this study, a polyclonal rabbit PTEN antibody (Y18 ab32199, Abcam, Cambridge, UK), a monoclonal rabbit ERG antibody (Y18 ab133264, Abcam, UK), a monoclonal rabbit SPINK1 antibody (Y18 ab207302, Abcam, UK), and a monoclonal rabbit TFF3 antibody (Y18 ab108599, Abcam, UK) were used. After dewaxing the tissue, antigen retrieval was performed for 20 min in PT-link (DAKO, Glostrup, Denmark) using High PH buffer. The slides were washed in phosphate-buffered saline before being blocked with 2.5% hydrogen peroxide. The sections were incubated for 30 min at room temperature with PTEN, ERG, SPINK1, and TFF3 (Ref CM 421C). The Dako Flex dual link detection kit (secondary antibody and DAB system K 8000, DAKO, Denmark) at a dilution of 1:100 was utilized for signal detection. Some IHC-stained tissue was lost during specimen cutting and staining; the remaining tissue was evaluated with 112 specimens for PTEN, 108 specimens for ERG, 104 specimens for SPINK1, and 114 specimens for TFF3.

**Figure 1:**
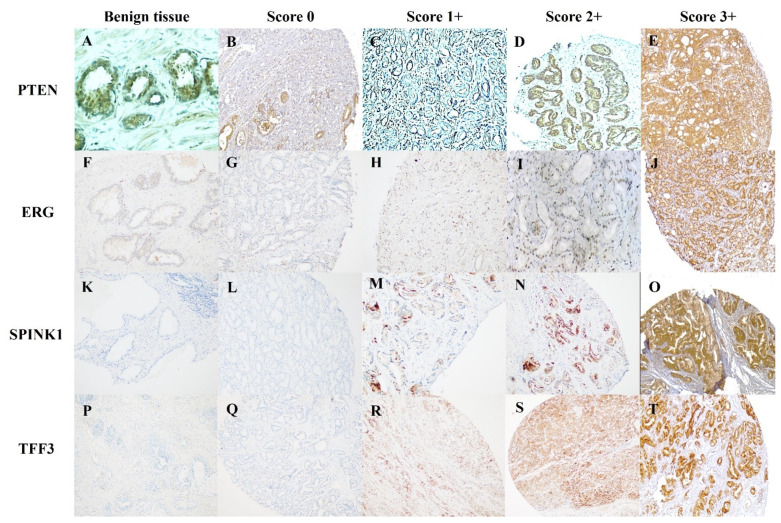
IHC for PTEN, ERG, SPINK1, and TFF3. Intact PTEN in benign tissue (**A**). PTEN loss in PCa, Score 0 (**B**) and Score 1 (**C**). Intact PTEN in PCa, Score 2+ (**D**), and Score 3+ (**E**). Negative ERG in benign tissue (**F**). Negative ERG in PCa, Score 0 (**G**), and Score 1+ (**H**). Positive ERG in PCa, Score 2+ (**I**), and Score 3+ (**J**). Negative SPINK1 in benign tissue (**K**). Negative SPINK1 in PCa, Score 0 (**L**), and Score 1+ (**M**). Positive SPINK1 in Pca, Score 2+ (**N**), and Score 3+ (**O**). Negative TFF3 in benign tissue (**P**). Negative TFF3 in Pca, Score 0 (**Q**), and Score 1+ (**R**). Positive TFF3 in PCa, Score 2+ (**S**), and Score 3+ (**T**).

PTEN, ERG, SPINK1, and TFF3 staining in the tumor was compared with stromal and endothelial cell reactivity, which was regarded as an internal positive control, while benign prostatic glands served as an internal negative control. The amount of staining was graded using a four-tiered system: 0 (negative), 1+ (weak), 2+ (moderate), and 3+ (intense) reactivity, as well as the proportion of positive tumor cells (Figure 1). Three expert pathologists (SAB, NA, and IIM) independently and blindly examined the immunostaining, and IHC scores for PTEN, ERG, SPINK1, and TFF3 expression were established. The overall agreement between separate reviewers was 95%, and in the rare cases where there was interobserver variability, the consensus was achieved by a collaborative microscopic review. We considered the scores 0–1 as ‘PTEN loss’ and 2–3 as ‘intact PTEN’. In regard to ERG, SPINK1, and TFF3 expression, 2+ to 3+ (moderate to intense) staining were treated as positive, and 0 to 1+ (negative to weak) staining were considered as negative. A multiplicative quick score was calculated as the product of intensity scores and the percentage of stained tumor cells.

Data were presented using numbers and percentages as well as means and standard deviations as appropriate. Chi-square test was used for comparison of proportions. Alpha level was set at 0.05.

## 3. Results

A total of 130 PCa cases were included in this study. The mean (SD) age at PCa presentation was 71.41 (10.94) years, and the mean (SD) of baseline serum PSA level was 67.07 (161.72) ng/mL. About one-third (29.2%, *n* = 38) of cases were GS 3 + 4(GG 2). However, 41.5% (*n* = 54) were GG5 according to the WHO-ISUP GG system. Only 47 PCa cases had a recorded pathological stage, among which twenty-six cases (55.3%) being classified as pathological stage 2, and only one case (2.1%) was classified as pathological stage 4 (Table 1).

The expression of IHC stains for PTEN, ERG, SPINK1, and TFF3 was correlated with patient age at presentation and preoperative PSA level. The expression of ERG and TFF3 was significantly correlated with patient age (*p*-value 0.011 and 0.019; respectively). The mean for preoperative PSA level was 117.45 ng/mL for PTEN loss (*n* = 46) and 36.6 ng/mL for intact PTEN (*n* = 58) (*p*-value 0.017). However, no statistically significant difference was found in the expression of PTEN and SPINK1 IHC stains and patient age at presentation. Furthermore, the expression of ERG, SPINK1, and TFF3 revealed no significant correlation with the preoperative PSA level (Table 2).

Among study participants, 48 cases (42.9%) exhibited a lack of PTEN expression (IHC scores 0 or 1), while the remaining cases (*n* = 64, 57.1%) showed intact PTEN expression (IHC score 2 or 3). In contrast, positive ERG was observed in 64 cases (59.3%), SPINK1 expression was observed in 6 cases (5.8%), and TFF3 staining was detected in 53 cases (46.5%) (Table 2).

A significant relation was observed between PTEN expression and ISUP Score which revealed a potential increased loss of PTEN expression as ISUP Score increases (*p*-value 0.019). Among cases with ISUP GG5, 55.3% showed PTEN loss, while 44.7% showed intact PTEN expression. On the other hand, 11.8% of ISUP GG1 showed PTEN loss and 88.2% showed intact PTEN expression. Similar statistical results were observed for the relationship between risk groups and PTEN (*p*-value 0.009) (Table 2).

As shown in Table 3, among all PCa cases (*n* = 104), 3.8% (*n* = 4) showed SPINK1+/ERG+ phenotype, 1.9% (*n* = 2) showed SPINK1+/ERG- phenotype, 56.7% (*n* = 59) showed SPINK1-/ERG+ phenotype, and 37.5% showed SPINK1-/ERG- phenotype (*n* = 39). Among ERG positive cases (*n* = 63), 6.3% were SPINK1 positive. Among SPINK1 positive cases (*n* = 6), 66.7% were ERG positive. The distribution of PCa cases was statistically significant by SPINK1 and TFF3 but not by SPINK1 and ERG or PTEN. Among the TFF3 positive cases, 11.5% were SPINK1 positive and among the TFF3 negative cases, 0% were SPNIK1 positive (*p*-value= 0.013). Among the SPINK1 positive cases (*n* = 6), all were TFF3 positive while among the SPINK1 negative cases (*n* = 98), 52 were TFF3 negative, and 46 were TFF3 positive.

## 4. Discussion

PCa is the most frequent male malignancy and the fifth greatest cause of cancer mortality in males worldwide [32,33]. In 2020, there were 1,414,249 newly diagnosed cases and 375,000 fatalities globally from this cancer [1,32,33,34,35]. PCa is the most commonly diagnosed malignancy in more than half of the world’s countries (112 out of 185) [36]. Malignancy in the prostate gland begins when cells of the prostate start to grow uncontrollably, typically accompanied by genetic and epigenetic changes that drive abnormal cell development. The most common changes that are seen in PCa are mutations in the PTEN, ERG, SPINK1, and TFF3 genes.

PTEN mutation in PCa has been associated with more aggressive behavior and poor prognosis. Several studies have reported a loss of PTEN in approximately 40–60% of PCa cases [37,38]. Findings of the current study are consistent with these results, as we observed PTEN loss in 42.9% of the evaluated cases, in which it is associated with a significant increase in tumor ISUP Score (*p*-value 0.019). In the current study conducted with PCa patients from the Middle East, the observed frequency of PTEN loss (42.9%) falls within the reported range in cohorts from Western countries but is higher than the rates reported in East Asian cohorts [37,38,39,40]. Similarities between our findings and those from Western countries may indicate similar biological behavior of the tumor, thus implicating the usefulness of results of clinical studies conducted in Western population for the therapeutic benefit of patients from the Middle East.

Chromosomal translocation resulting in the generation of the TMPRSS2-ERG fusion gene has been found in 50% of PCa [41]. In a study by Aldaoud et al., the prevalence of ERG expression was evaluated in Jordanian-Arab PCa patients [42]. It was discovered that 33% of analyzed specimens had ERG expression, which is lower than in the Western PCa population but greater than in Asian cohorts. In our study, we observed ERG expression in 62.2% of our PCa cases, which is higher than the results reported by Bismar et al., where the rate was found to be 41.5% [43].

Further studies are needed in diverse racial populations to investigate the influence of genetic, epigenetic, lifestyle, and environmental factors in more detail. These studies will help determine the causes of some of the variations in the frequency of PTEN loss and ERG expression in PCa between populations from Western Europe, North America, East Asia, and Jordanian patients [42].

In a study conducted by Terry et al. in 2015, it was found that TFF3 expression is present in 66% of PCa cases with observed correlation between patient age and TFF3 expression [44]. Our results reveal that 46.5% of PCa cases show TFF3 overexpression, and TFF3 overexpression is seen in the younger age group (*p*-value 0.019).

Overexpression of SPINK1 has been linked to poor prognosis in several cancers including PCa [45]. In our study, we identified SPINK1 expression in 5.8% of cases. In contrast, a study conducted by Räsänen et al. reported presence of SPINK1 in 10% of their cohort [46]. Moreover, Terry et al. [44], observed that every PCa with SPINK1 positivity had also TFF3 positivity which is concordant with our results. Our findings reveal that SPINK1 expression was predominantly observed in a subgroup of cancers that expressed TFF3 (*n* = 6/6, *p*-value 0.013 highlighting the potential significance of assessing both TFF3 and SPINK1 statuses in order to stratify the risk of PCa patients. Finally, the evaluation of ERG, TFF3, and SPINK1 could be an attractive approach to determine both tumor heterogeneity and PCa subtypes.

## 5. Conclusions

This is the first study to look at the status of the PTEN, ERG, SPINK1, and TFF3 genes in a Jordanian Arab population. Loss of PTEN has been linked to more aggressive prostate cancer with high GSs/GGs. SPINK1 expression was predominantly observed in a subgroup of cancers that expressed TFF3. Our results call for screening these biomarkers for grading and molecular subtyping of the disease.

## Figures and Tables

**Table 1:** Clinicopathological variables and biopsy score among Northern Jordan prostate cancer patients.

Age at presentation (Years) Mean (SD) Median (IQR)			
	71.41 (10.94)		
	72.0 (13)		
Baseline serum PSA (ng/mL) Mean (SD) Median (IQR)			
	67.07 (161.72)		
	12.6 (42.79)		
Gleason Score		Number	Percent
	3 + 3	20	15.4
	3 + 4	38	29.2
	4 + 3	7	5.4
	3 + 5	1	0.8
	4 + 4	10	7.7
	4 + 5	28	21.5
	5 + 4	5	3.8
	5 + 5	21	16.2
Total		130	100.0
ISUP Score (Grade Group)			
	1	20	15.4
	2	38	29.2
	3	7	5.4
	4	11	8.5
	5	54	41.5
Total		130	100.0
Pathologic stage			
	2	26	55.3
	3	20	42.6
	4	1	2.1
Total		47	100.0

**Table 2:** Distribution of study participants by mean age, mean PSA, ISUP Score, and by PTEN, ERG, SPINK1, and TFF3 IHC.

			**PTEN**		**Total**	**ERG**		**Total**	**SPINK1**		**Total**	**TFF3**		**Total**
			**Loss**	**Intact**		**Negative**	**Positive**		**Negative**	**Positive**		**Negative**	**Positive**	
**Mean age at presentation**			70.51 (*n* = 47)	70.72 (*n* = 60)		73.88 (*n* = 42)	68.49 (*n* = 61)		70.89 (*n* = 90)	68.5 (*n* = 4)		72.82 (*n* = 60)	68.08 (*n* = 49)	
*p*-value			0.920			0.011			0.62			0.019		
**Mean Preoperative PSA (ng/mL)**			117.45 (*n* = 46)	36.60 (*n* = 58)		80.84 (*n* = 39)	69.30 (*n* = 61)		77.47 (*n* = 92)	56.84 (*n* = 4)		67.03 (*n* = 57)	76.02 (*n* = 48)	
*p*-value			0.017			0.751			0.820			0.832		
**ISUP ** **Score (Grade Group, GG)**	GG1	Number	2	15	17	5	12	17	16	1	17	8	11	19
		Percent	11.8%	88.2%	100%	29.4%	70.6%	100%	94.1%	5.9%	100.0%	42.1%	57.9%	100%
	GG2	Number	11	21	32	15	18	33	30	1	31	16	16	32
		Percent	34.4%	65.6%	100%	45.5%	54.5%	100%	96.8%	3.2%	100.0%	50.0%	50.0%	100%
	GG3	Number	3	2	5	1	4	5	4	0	4	4	2	6
		Percent	60.0%	40.0%	100%	20.0%	80.0%	100%	100.0%	0.0%	100.0%	66.7%	33.3%	100%
	GG4	Number	6	5	11	6	5	11	10	1	11	6	5	11
		Percent	54.5%	45.5%	100%	54.5%	45.5%	100%	90.9%	9.1%	100.0%	54.5%	45.5%	100%
	GG5	Number	26	21	47	17	25	42	38	3	41	26	20	46
		Percent	55.3%	44.7%	100%	40.5%	59.5%	100%	92.7%	7.3%	100.0%	56.5%	43.4%	100%
Total		Count	48	64	112	44	64	108	98	6	104	61	53	114
		Percent	42.9%	57.1%	100%	40.7%	59.3%	100%	94.2%	5.8%	100.0%	53.5%	46.5%	100%
*p*-value			0.019			0.563			0.971			0.790		
**Risk group**		Low	16	38	54	21	34	55	50	2	52	28	29	57
			33.3%	59.4%	100%	47.7%	53.1%	100%	51.0%	33.3%	100%	46.7%	53.7%	100%
		High	32	26	58	23	30	53	48	4	52	32	25	57
			66.7%	40.6%	100%	52.3%	46.9%	100%	48.0%	66.7%	100%	53.3%	46.3%	100%
*p*-value			0.009			0.696			0.678			0.574		

**Table 3:** Distribution of study participants by SPINK1 and by ERG, TFF3, and PTEN.

		SPINK1					
		Negative		Positive		Total	*p*-Value
		Number	percent	Number	percent	Number	
ERG	Negative	39	95.1%	2	4.9%	41	0.557
	Positive	59	93.7%	4	6.3%	63	
Total		98	94.2%	6	5.8%	104	
TFF3	Negative	52	100%	0	0%	52	0.013
	Positive	46	88.5%	6	11.5%	52	
Total		98	94.2%	6	5.8%	104	
PTEN	Loss	42	97.7%	1	2.3%	43	0.178
	Intact	53	91.4%	5	8.6%	58	
Total		95	94.1%	6	5.9%	101	

## Data Availability

The data presented in this study are available within the article.

## Abbreviations

PCa	Prostate cancer
TMA	Tissue microarray
PTEN	Phosphatase and TENsin homolog
ERG	Erythroblast transformation-specific–related gene (ETS-related gene)
SPINK1	Serine protease inhibitor Kazal-type 1
TFF3	Trefoil Factor 3
RP	Radical prostatectomies
TURP	Transurethral resections of prostate
GS	Gleason Score
GG	Grade Group
ISUP	International Society of Urological Pathology
PSA	Prostate-Specific Antigen
IHC	Immunohistochemistry
